# Disconfirmation of Taste as a Measure of Trust in Brands: An Experimental Study on Mineral Water

**DOI:** 10.3390/foods11091276

**Published:** 2022-04-28

**Authors:** Elena Kokthi, Ledia Thoma, Reka Saary, Aniko Kelemen-Erdos

**Affiliations:** 1 Faculty of Biotechnology and Food, Agriculture University of Tirana, P.C. 1000 Tirana, Albania; ekokthi@ubt.edu.al; 2 Faculty of Economics and Agribusiness, Agriculture University of Tirana, P.C. 1000 Tirana, Albania; ledia.thoma@ubt.edu.al; 3 Keleti Károly Faculty of Business and Management, Óbuda University, P.C. 1084 Budapest, Hungary; kelemen.aniko@kgk.uni-obuda.hu

**Keywords:** brand equity, brand management, Expectation–Disconfirmation Theory, grounded theory, asymmetric disconfirmation, bottled mineral water

## Abstract

The underlying factors of the purchase decision process of bottled mineral water have been a less studied area. The typically related attributes of consumer judgement in the case of low involvement can vary widely, ranging from price sensitivity to habits. However, assessing the role of brand reputation and trust from a sensory perception perspective is not a common approach. This paper examines the impact of trust on consumer value judgements for a frequently consumed beverage such as mineral water. Combining trust and sensory attributes with the Expectation–Disconfirmation Theory (EDT) framework provides insights into the weight of taste, trust and reputation in product evaluation. A tasting experiment was carried out using a representative systematic random sampling method. A mixed method was applied; EDT was used to analyze quantitative data and grounded theory methodology was performed in the case of qualitative data. Results indicate complete assimilation for the most preferred brand and negative contrast for less well-known brands. It can be stated that the applied methodology is suitable as a certain kind of trust measurement and also can function particularly well as a reinforcement and complement to other methodologies (e.g., neuromarketing methods). This study suggests that brand names positively influence value judgment. Origin bounded brands compared to imported brands can help companies mitigate trust issues in developing countries as they convey a particular reputation, which helps reinforce trust.

## 1. Introduction

Safety incidents in the food system have a deleterious effect on consumer trust [1]. Consumer trust is an essential aspect in the functioning of any market but mainly in the food and drinks sector [2]. Food scandals, such as the bovine spongiform encephalopathy outbreak in the 1990s, the ‘horsemeat scandal’ in 2013, dioxins in food in Belgium in 1999, and the detection of mad cow disease in Britain affect consumers’ trust [3]. In addition to food incidents, increased sophistication and globalization of food markets are accompanied by the distancing of the consumer from the production source. Similarly, this augmented complexity and distance have contributed to a decline in trust and simultaneously increased the importance of trust.

This is a challenging issue in developing countries such as Albania, where consumers lack trust in institutions, especially those linked to regulatory systems related to food [4]. Trust is essential in individuals’ food purchasing decisions and understanding the factors that stimulate and mitigate consumers’ trust in food, to inform the public and business sector, is necessary for both developed and developing countries. The trust concept is analyzed mainly in social sciences because of its substantial relationship with development in general and socioeconomic development in particular [5,6]. No single consensual definition is agreed on trust, and several nuances persist in the academic debate [7,8]. However, trust is essential for cooperative behavior and solving collective action problems [9]. In addition, rational choice scholars implicitly or explicitly equate trust with simple institution-induced expectations [10]. Similarly, in the entrepreneurial context, a firm owner expects a business partner to act in their interest or take such interests into account, which is also an expectation issue [8].

According to the paper review of consumers and trust, Hobbs and Goddard [7] mention four broad categories of trust as follows: (1) institutional trust (trust in regulatory systems), (2) generalized trust (measured through the general trust that people have in others), (3) calculative trust (individuals behaving in such a way that does not cause harm for their interest, and (4) finally relational trust that derives from the cumulative experience between the trustee and trustor. The last one derives from familiarity and experience [11,12]. The four mentioned categories of trust had been analyzed in the food context, showing an impact on specific consumer decisions. Ding and his co-authors [13] analyze the relationship between generalized trust and consumer behavior, showing that in the case of health-risk related functional food, trusting people are more likely to buy them. Although in the case of the environmental footprint related to food, generalized trust does not impact German consumer choices [14]. Peters et al. [15] also show that institutional trust impacts attitudes toward biotechnology in USA consumers and not in German ones. Similarly, Siegrist and Hartmann [16] show that trust in the food industry directly influences the acceptance of cultured meat. Consumers who trust the food industry are more likely to buy functional foods than consumers who do not [17].

Consequently, trust has an influence on customer choice and perceived quality, and therefore, on customer satisfaction [18]. Other studies show the interrelationship between trust and consumer risk perceptions in food choices; Janssen and Hamm [19] point out that German consumers show low trust in the European Union (EU) mandatory logo on organic products vs. German logos. In comparison, Albanian consumers show the contrary [4]. When dealing with consumer and trust issues, researchers have reiterated the importance of institutional trust by jointly merging public with private activities. Indeed, the lack of trust in public institutions in the food industry erodes consumer confidence even in private institutions [20]. In this framework, brands represent private institutions that might reduce the risks in food choices induced by the lack of trust in public institutions. In the definition of Lin and Nugent [21] (p. 2037), institutions are defined as “*a set of humanly devised behavioral rules that govern and shape the interactions of human beings, in part by helping them to form expectations of what other people will do*”. In this vein, brand processes and activities shape human interactions around a product and service and generate expectations.

Consequently, strong brands can mitigate the effect of low trust in institutions and consumer decisions [22]. Origin bounded brands (OBBs) can mitigate through reputation the negative effect of low trust in institutions [23,24,25]. However, whether in people, organizations or marketing constructs such as brand, trust is not an immutable attitude, and most changes in it are in a negative direction due to the greater salience of negative information [26]. Thus, monitoring the trust levels in public or private companies is particularly relevant nowadays, given the amount of information that the individual has to process daily. In that regard, customer-based brand evaluation processes from a trust perspective play a pivotal role. This paper presents trust as an inherent part of the brand and uses expectations (E) as a proxy to evaluate it. The rationale of this study is based on the model of consumer-derived brand equity (BE), with a focus on perceived quality as a dimension of BE. The brand’s share in the product’s overall evaluation is analyzed through the difference between sensory perceptions of the product in blind tests and when brand information is available to the consumer. This analysis is based on the Expectation–Disconfirmation Theory (EDT), which helps us appraise the level of trust generated by the credence attribute (the brand). The disconfirmation of taste will indicate trust in four experimented mineral water brands.

The paper is organized as follows: the first section includes a brief literature review of EDT, BE measurement and trust operationalization. The second section presents the general experimental approach of EDT. The experimental design and the results are presented in the following section. A discussion of the findings concludes the paper.

## 2. Literature Review

In markets where products and services have become similar, with no significant functional differences and consumer choices are more and more influenced by emotional aspects rather than rational thinking, experiences have surfaced as the primary form of differentiation [1]. Marketing academics and practitioners have acknowledged that consumers look for brands that provide them with unique and memorable experiences [2]. In this vein, from the consumer viewpoint, brands are relationship builders, and the sensory information that they convey affects satisfaction, trust and loyalty [2]. The sensory perception of consumers has explicit and implicit impacts on brand evaluation. Haas and her co-authors show that the implicit factors have a notable effect on explicit characteristics and brand experience and do not contradict each other [22]. In the same vein, non-physical, intangible factors can affect consumer sensory perceptions.

Consequently, the customer believes more in the brand than the objective features in the absence of information. Many studies view brand trust as central and conceptualize it as a notable factor in the firm’s success [27]. Brand trust is viewed as a long process that can occur by considering consumer experiences. Therefore, brand trust can be discussed as a cognitive component that may induce an emotional response and expectations [28].

Several authors have analyzed the impact of product credence attributes such as brand, advertisement, packaging, label, price and origin on sensorial expectations [29,30,31,32,33,34,35,36,37,38,39,40]. The application of neuromarketing tools is also a common procedure in this research field [41,42]; however, the reliability of these methods is not yet widely accepted [43]. Scholars, as mentioned earlier, have used Expectation–Disconfirmation Theory (EDT) to assess the influence of credence attributes on the consumer evaluations of several food products. However, to the authors’ knowledge, this theory was not used previously to measure trust based on brand expectations.

Disconfirmation of expectancies refers to the difference between expectations and objective quality, or in other words, the real performance of a product [29,30,44,45,46,47]. According to Lee and his co-authors [48], expectations create the baseline for satisfaction—if disconfirmation occurs, customer satisfaction will be higher or lower than the baseline level. Disconfirmation is positive when product performance exceeds expectations and vice versa [45]. In addition, disconfirmation can be asymmetrical when positive and negative disconfirmations are not of the same size. Thus, analyzing the brand’s perceived quality and expectations, its strength from a consumer perspective and the level of trust the latter confers to them can be evaluated.

When consumers taste a food product, their perception is often biased by preconceived ideas in product evaluation. Schifferstein [44] identifies a set of three alternative ways to isolate sensory from non-sensory preferences: (1) blind testing with a product, which provides experience attribute information; (2) expectation testing (E), which permits the collection of credence (accumulated trust) attribute information; and (3) full information testing (involving the provision to the consumer of experience and credence information regarding the product). The differences between the scores measured using these three tests can be used to measure BE and the trust score for the specific brand from the consumer perspective.

Full information test score (F) − Expectation score (E) = Degree of Disconfirmation

Expectation test score (E) − Blind test score (B) = Degree of Incongruence (reputation)

Full information test score (F) − Blind test score (B) = Degree of Response shift (trust on brand information)

Following the logic of EDT, a strong brand can significantly improve the full evaluation of a product—i.e., the type of evaluation that combines experience and trust in brand information. A strong brand prevails in a full evaluation situation; that is, the brand significantly affects the full product evaluation compared to a blind tasting scenario [30]. In contrast, weak brands may prevail in blind evaluations, i.e., brand reputation does not significantly affect the full product evaluation compared to blind tasting. Assimilation theory attempts to explain this behavior [49] by positing that assimilation occurs when unconfirmed expectation-related discrepancies are assimilated by aligning perceptions with expectations [29,44,49,50]. When assimilation is absent, this suggests that a brand name does not interfere with sensory perception in the overall product evaluation and that the blind score should be equal to the full information score [29,51]. A contrast effect (positive or negative) occurs when a change of product evaluation in the full information scenario moves in the opposite direction to the expected value information (ibid.). Contrast is more likely to occur with well-known products [52], since these tend to rely more on experience information than on credence (i.e., non-sensorial) information [51].

Another factor involved in BE is asymmetric disconfirmation. Schifferstein et al. [31], and Anderson and Sullivan [51] discovered that consumer satisfaction (CS) is more sensitive to negative disconfirmation than positive disconfirmation. Under these circumstances, product managers focus more on avoiding negative performance perceptions than on enhancing positive performance perceptions (ibid.). Thus, measuring this aspect is quite important in product and brand management. Several studies show the relationship between trust reputation and sustainability, especially with the increasing importance of community marketing [53]. Even though trust and reputation are a chicken–egg issue, the former allows the reputation to exist. By promising and meeting the expectations over time, organizations build trust.

**Figure 1:**
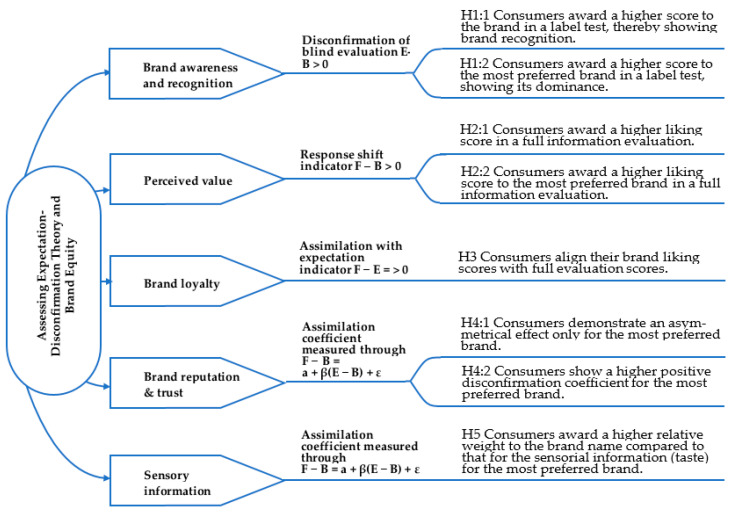
Mapping brand equity with Expectation–Disconfirmation Theory. Note: Expectation score (E); Blind test score (B); Full information test score (F); B1, B2, B3, B4: conducted brands; α: Level of assimilation (proportion of the response shift over the degree of incongruence); β, ε: function coefficients. Source: Authors’ construction.

In this present study, BE with EDT indicators (presented in Figure 1) has been operationalized to assess the value of four brand reputation–trust instances, according to consumer perceptions. Following Aaker’s [54] BE model, three indicators are proposed: (1) brand awareness, examined through brand recognition and brand dominance; (2) perceived value; and (3) brand loyalty, measured by evaluating the relative share of brand influence in the overall product evaluation, and through the asymmetrical effect of expectations on satisfaction. Brand awareness refers to whether consumers can recall or recognize a brand or simply whether they know about a brand [55]. Brand awareness and familiarity foster customers’ recollection and recognition of brands [56]. The brand name activates a memory [54], and brand knowledge is linked to the brand name, culminating in brand equity [54,57]. However, incongruent, suggestive brand names can cause unintended detrimental effects related to product choice [58].

Several authors [59,60] have confirmed the positive association between brand awareness and BE. In addition, brand awareness is an important indicator because it mediates between brand loyalty and BE. In the present study, brand awareness is expressed in H1.1 and H1.2.

The second indicator considered in BE evaluation is perceived quality. Perceived quality is defined as a consumer’s evaluation of a brand’s overall excellence based on intrinsic (taste) and extrinsic cues (brand name); for that reason, perceived quality in itself is a compound construction [54]. In addition, perceived quality also generates value for consumers by increasing consciousness and providing them with a reason to buy and differentiating one brand from competing brands [61,62]. This suggests that perceived quality is one of the main elements of BE and an essential factor in evaluating brand equity. In marketing, the construct of perceived quality has been widely acknowledged as the primary driver of purchase intention [63]. When the informational process includes mention of the brand of the product, it has been reported that consumers who were formerly indifferent in terms of their preference for products in the blind test demonstrate a strong sensory preference for the most preferred brand. One of the theories that explain this behavior is Assimilation Theory. According to this theory, assimilation occurs when unconfirmed expectations discrepancies are assimilated by aligning perceptions with expectations [29,50,64]. According to Nobel Prize winner in psychology Daniel Kahneman, this behavior is linked with a well-known confirmation bias misconception. Individuals tend to interpret new information to become compatible with their existing beliefs and convictions. The more nebulous the pieces of information are, the higher the confirmation bias is. In that regard, trust has a vital role to play in the mitigation of this decision-making fallacy.

When the brand does not affect the sensory valuation, α equals zero, whilst the irrelevance of sensory characteristics in an overall evaluation leads to a score of one. When assimilation is absent, the brand does not affect the sensory perception in the overall evaluation of the product, and the blind score should be equal to the full information score. Thus, α should be equal to zero. The hypotheses and respective indicators used to test perceived quality are presented in Figure 1. The third indicator analyzed in the present study is brand loyalty. Aaker [54] defines brand loyalty as a constructive mindset about a brand that leads to constant purchasing over time. The author also argues that brand loyalty is an essential and valuable element in consumer evaluation because loyalty can generate profit. In this study, a positive or a negative assimilation effect is used to measure brand loyalty whenever the change of product evaluation in a full information condition is in the same direction as the expected value. Consumers will tend to assimilate their evaluation to their expectancy of the most preferred brand, showing brand loyalty (see H3). If the disparity between expectations and performance is to be perceived, the consumer will accept it because of the trust placed in the brand.

Assimilation can be measured as the proportion (α) of the response shift over the degree of incongruence:

(1) α=(F−B)/(E−B) with 1≥ α ≥0

Note: Expectation score (E); Blind test score (B); Full information test score (F).

The contrast effect, which may be positive or negative, occurs when the change of product evaluation in the full information condition changes in the opposite direction to the expected value.

From this perspective, the combination of Aaker’s BE model with EDT can be used to measure the strength of brand trust–reputation in the market from the customer perspective.

## 3. Material and Methodology

### 3.1. Product Selection

Four bottled mineral water brands are evaluated in this research. Market share is one of the most common selection criteria for such studies. However, due to the lack of official data about the market share/consumption of different bottled water brands in Albania, a preliminary study was undertaken to identify the most commonly consumed water brands in Tirana (Albania’s capital). A set of 100 vox-pop (“voice of the people”) interviews were implemented. Thirty-six percent of respondents regularly consume Lajthiza (Brand 1), 19 percent drink filtered tap water, 17 percent drink Tepelena (Brand 2), 12 percent Qafshtama (Brand 3), and 3 percent Acqua Panna (Brand 4). The remaining 13 percent did not mention any specific brand. Brand 1 (Lajthiza) and Brand 3 (Qafshtama) refer to the names of springs in the Albanian mountains from which the water is sourced. Brand 2 is origin-bounded because the water comes from a well-known region for mineral water in Albania (named Tepelena), while Brand 4 is an imported product (Italian origin). The latter is included in the BE analysis for two reasons—first, to create a familiar or quasi-market situation for consumers in terms of offering domestic and imported products and second, to analyze the trust–reputation issue through the BE–EDT approach. The Albanian bottled brands’ market prices are the same, ALL 50 (about EUR 0.42)/1500 mL in consumer markets, while the imported brand is about 50% more expensive. Since the scope of the study is focused just on brand, we have not considered other characteristics.

### 3.2. Research Design and Sampling

From the senses, gustatory features have the most notable influence on purchase intention and consumers’ purchase decisions [65]. Thus, the taste experiment approach of this study can be thereby supported.

The experimental design was developed using a three-point situational framework: (1) Blind test with the product, (2) Expectation test, which included providing only extrinsic (brand) information, and (3) Full information test (provision of experience and credence attributes regarding the product). The blind test enables the assessment of the explicit cues that are objective and conscious, while the implicit cues that are credence attributes can be conducted in the brand and full information test. The research was carried out with the approval of the Institutional Ethics Committee of the Agricultural University of Tirana (project No.: 18/1025/2020). Respondents participated anonymously and of their own free will, following the committee’s instructions.

The research was conducted in Tirana, where nearly one-third of the population resides (about 900,000 inhabitants from a total national population of 2.9 million). Participants were pre-screened according to primary purchasers who regularly buy bottled mineral water within the family. Systematic random sample selection was applied using the Tirana phone book; this resulted in a representative random choice sample of the city’s entire population. Any nonresponse was replaced with the following number of the phone book. Participants who agreed to participate in the experiment were invited to participate according to their availability. The tasting experiment was developed on the Agricultural University of Tirana premises. It was organized on ten consecutive days with 25 participants each day.

The applied experimental procedure was as follows. First, the four products were tasted in blind information conditions. Respondents were asked to assess the products using five-point hedonic scores, express their liking scores, and explain their evaluation by answering an open-ended question. After each tasting, participants ate some bread to cleanse their palettes. Next, the labels for each mineral water brand were presented on a piece of paper, and the only information provided was the brand name. Second, the order in which the labels were presented was modified compared to the first stage (the blind test). Finally, the same subjects tasted the products in the full information conditions. The differences in consumer product perceptions between full and blind information settings were assumed to reveal the value of the brand and its trust–reputational strength from the consumer perspective.

Partially for data collection, but primarily in data processing, mixed methods [66] were used. The data collected through the applied hedonic scales were processed using statistical methodologies and assimilation coefficient (EDT) calculation. At the same time, responses to open-ended questions related to each testing phase were analyzed using grounded theory as a qualitative research methodology. Adopting the grounded theory (GT) methodology helped us understand the participants’ deeper motivations. Following the guidance of GT by Strauss and Corbin [67], firstly, the answers were transcribed, and initial open codes were formed using the original expressions applied by the respondents. Secondly, axial coding ensured the finding of connections among the particular variables. Thirdly, selective coding provided a screened picture of the main features. Theoretical codes were used to explore the factors underlying the role of sensory and credence attributes, particularly brand, in consumer decisions.

### 3.3. Hedonic Scale Selection

Sensory brand experience is evoked directly by sensory brand-related stimuli. Consequently, establishing unique and robust impressions in consumers’ minds through five human senses (sight, hearing, touch, smell and taste) is critical in the brand management process [68]. Sensory analysis of food examines the organoleptic properties that are feasible with the senses and is generally undertaken through descriptive, discriminative and hedonic testing [69]. In this present study, hedonic tasting is applied. In consumer research, hedonic scales are well tried and tested for capturing liking data [70]. The nine-point hedonic scale is probably the most useful sensory method [71]. However, the original nine-point scale does not necessarily apply to the translations of the scale, and the verbal anchor’s discriminative ability is not always confirmed [72,73]. In comparing the nine-point hedonic scale used between Americans, Chinese, Koreans and Thai, Thai respondents use the nine-point hedonic scale differently from American respondents. They use a smaller range of the nine-point hedonic scale than Americans [73]. The nine-point hedonic scale is not used in food research in Albania because of the difficulty of the verbal translation of the scale. Other studies in Albania have shown the successful adaptation of the five-point Likert scale in liking hedonic scores in tasting experiments with cheese [38], different Cola brands [39] and other food attributes [39]. Applying the five-point liking score in the studies mentioned above showed a reliable and discriminative feature. In this study, a five-point hedonic score (1 = I don’t like it at all, 5 = I totally like it) was adopted where the participants expressed their liking scores for the sensory attributes of the four tested mineral waters and their respective brands. In order to explore the reasons for consumer decisions, qualitative associative questions were compared with quantitative ones in every stage to highlight the main features that were taken into account in consumer choices. The evolving qualitative associations will also help to examine the hedonic scoring reliability.

### 3.4. Hypotheses and Approach

Through Hypothesis 1, brand awareness of the four brands incorporated in the study can be identified; H1.1—consumers express a higher score for the brand in a labelled test, thereby showing brand recognition and reputation. Mean score *t*-tests were applied to each difference in information to detect significant differences: (1) Expectation − Blind (E − B); (2) Full information − Blind (F − B); and (3) Full information − Expectation (F − E). A statistically significant difference depicted in (E − B > 0) shows that the brand attribute affects consumer preference, indicating recognition and reputation. In addition, paired *t*-tests were carried out to identify differences in liking scores between the four selected brands; H1.2—consumers award higher scores to the most preferred brand in a labelled test, showing its dominance. The second indicator used to examine BE was perceived value through the response shift. This indicator measures to what extent brand name and taste affect the liking scores of the products. The higher the value, the higher the brand evaluation and the stronger its equity (F − B). In addition, a statistically significant *t*-test concerning the response shift (F − B) between the four brands revealed those with higher BE; see Hypotheses 2.1 in Figure 1.

The third indicator used to examine BE was brand loyalty; the following hypothesis was tested through the assimilation theory (AT) indicator (H3.—consumers align their brand liking scores with full evaluation scores for the origin-bounded and imported products due to higher reputation). Full assimilation takes place when F − E = 0. That is the case when a consumer might not be totally satisfied but aligns their full information evaluation with the liking score yielded from the brand, showing brand loyalty. The assimilation coefficient was the fourth indicator that assessed BE through brand reputation and trust. Four regression slopes (F − B = α + β (E − B) + ε) were plotted to measure the relative weight of brand reputation in the overall evaluation of the product. If α < 0.5, the brand name has a lower product evaluation weight than the taste. The following hypothesis was tested in H4. Consumers award a higher relative weight to the brand name compared to that for the sensory information (taste), for the most preferred brand due to higher trust and reputation and H4.1. The brand’s share in the overall evaluation of the product is higher in the EU imported products due to higher trust in EU institutions.

## 4. Results

### 4.1. Quantitative Results according to Expectation–Disconfirmation Theory

A total of 250 product evaluations were completed, of which 230 were valid (20 participants’ evaluations were not considered in data analysis due to the lack of clarity in writing the liking scores). In Table 1, the demographic characteristics of participants are presented.

The means of the consumer scores obtained in the three scenarios are shown in Table 2. The results reflect, to a certain extent, the brand selection criteria. The preference revealed in the vox-pop interviews was confirmed in the research for the leading brand, Lajthiza (Brand 1), which scored the highest in all three evaluation phases.

Concerning the first indicator of brand equity, a statistically significant difference between the scores awarded in the blind condition and brand information stage was found for Brand 1, bottled mineral water sourced from the mountains, and Brand 4, the imported product (see Table 2). No difference was found for the other waters in terms of (E-B), which scored lower than Brands 1 and 4. The paired *t*-test comparisons between Brand 1 and Brand 2 show that consumers scored Brand 1 higher in the three situations (blind, brand, and full information). The same result was obtained when comparing Brand 1 to Brand 3. Brand 1 scoring is statistically higher than for Brand 3. Comparing Brand 1 with the imported product (Brand 4) shows that the products were scored the same in the blind and brand scenarios.

Pair comparisons of liking scores in the blind conditions show the dominance of Brand 1 (which is an Albanian brand, B1_mean_ = 3.5) and the imported brand (B4_mean_ = 3.4). The same results are observed when brand information is made available to the consumers; B1 and B4 are still the most preferred (B1_mean_ = 3.7 and B4_mean_ = 3.6). No significant differences are observed between B1 and B4 in both the blind and brand evaluation, meaning that the imported brand is equally as preferred as the most popular Albanian brand. However, the situation changes in the full information scenario; B1_mean_ = 3.8 and B4_mean_ = 3.4. Consumers have revised their evaluation of the imported brand by lowering it. The high expectations generated from the imported brand are linked to the level of trust that consumers confer in it. Interesting results are observed when comparing B2, which represents an OBB, with the imported brand, B4. In the blind condition B2_mean_ = 3.1; B4 _mean_ = 3.4 and in the brand evaluation condition B2_mean_ = 3.2; B4_mean_ = 3.6; consumers prefer the imported brand rather than the OBB. Thus, the imported brand scored higher in terms of taste and brand. These results reflect the lack of trust in the OBB. When information is completed (B2_mean_ = 3.4; B4_mean_ = 3.4), consumers revise their evaluation and lowers the score given to the imported brand. EDT shows that consumers make the difference between OBB and imported brands, showing that the consumer initially evaluated the imported brand positively because of less perceived risk in the imported product. However, when the information is completed, the reputation of the OBB influenced the liking score of the imported product, which engendered contrast behavior. Other research shows that reputation can enhance trust [74,75].

The general effect of the three information situations is also analyzed. The general liking scores of the four considered brands are as follows. The general liking score of the brands in the blind condition GBB_Mean_ = 3.3. (General brand evaluation in the blind full information evaluation.) The general liking score of the brands in the expectation condition GBE_Mean_ = 3.4. (General brand evaluation in the brand full information evaluation.) Finally, the general liking score of the brand in the full GBF_Mean_ = 3.3. (General brand evaluation in the full information evaluation.) Pair comparisons (E–B_Mean_ = 0.15, *p* (value) = 0.004); F–B_Mean_ = 0.06^ns^; F–E_Mean_ = −0.08^ns^) show that brand information is important. However, the weight of brand (F − B = α + β(E − B) + ε) in the overall evaluation of the mineral water is β = 0.32 and R^2^ = 0.35. In conclusion, the role of the brand in the overall evaluation of mineral water is low. These results confirm that EDT can give insights into brand reputation in the Albanian market. The lack of trust in food systems monitoring safety issues can be mitigated by brand reputation. However, in the long term, reputation is built on the belief that the company will do the right thing and deliver its promises. By promising and meeting the expectations over time, organizations can build trust and mitigate the negative effect of the lack of trust in the overall institutional settings. The relationship between reputation and trust can contribute to sustainability in organizations.

### 4.2. Qualitative Results according to Grounded Theory

The dimensions of mineral water evaluation have emerged in every research stage (blind, brand, and full) by collecting the associations made to taste, brand and complete information, resulting in more sophisticated taste descriptions in the second and third round tests. The number of attributes has notably increased from 12 through 22 up to 30 features that interpreted taste. Thus, evaluations shifted from physical product characteristics to additional associations in the brand test in the second round. However, the brand name alone could evoke restrained associations without its design, still emphasizing physical characteristics. The third, the full information phase, provided added value through brand equity.

**Figure 2:**
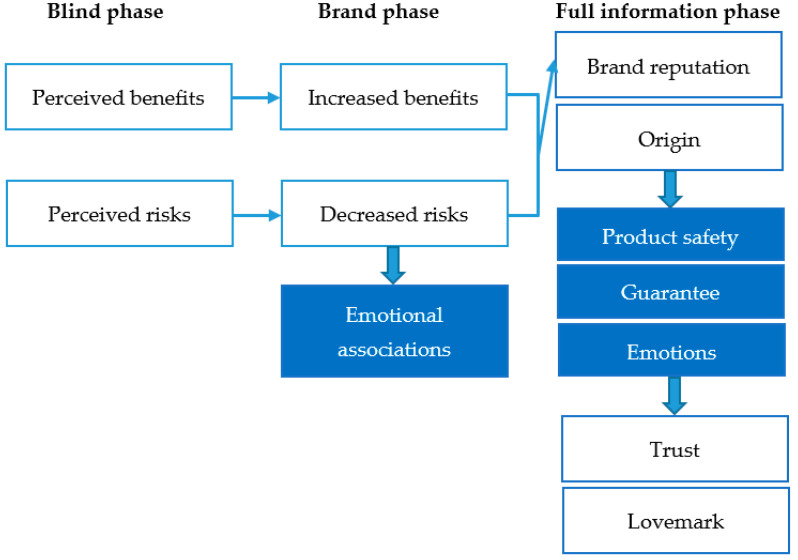
The role of brand reputation and design in consumer preferences regarding mineral water consumer decision.

The dimensions that emerged by using the grounded theory methodology are highlighted in Figure 2. The first phase could define the objective features as perceived benefits and risks. The main initial features in the first phase were sensory attributes that were related to taste and described with pairs of contrasts such as “heavy” and “light”, or “dense”, or “sweet” and “bitter”. Uncertainty occurred in the use of grammatical structures like “maybe”, “probably”, and “seemed to”. Thus, the first phase could theoretically be coded as perceived benefits and risks. In the brand phase, perceived benefits have increased, and perceived risks have clearly reduced. Negative associations decreased overall, and words expressing emotions such as “enjoy” have appeared. The full information phase shows that brand reputation could support product evaluation. In the full information phase, uncertainty decreased significantly.

Consequently, full brand information contributes to providing added value. Emotional associations have increased moderately in the third phase. It can be assumed that emotional associations could not become significant because of the product type. However, features characterizing credence attributes have appeared only in the third phase. The origin bound was emphasized by words such as “origin”, “imported”, “well-known”, “springs” and institutions”, and even their underlying factors like “safety”, “healthy”, “hygienic” and “trust”. The result that health issues were mentioned only in the third phase was surprising.

## 5. Discussion

Researchers have shown that, in the food context, brand trust is positively associated with consumer confidence in brand quality and safety, mainly via trust in the distribution chain [1,76]. In developing countries like Albania, where the institutional environment and related controls still need improvement, brand names induce more trust among consumers. In this case, internationally well-known brands such as Acqua Panna are considered safer than national brands. However, these findings need further empirical verification as customers may not recognize international brands and trademarks [1]. Nevertheless, especially in Albania, international brands are highly valued [76]. However, the situation changes in the full information condition, revealing a stronger preference for the domestic product. In the case of the domestic brand, the name represents an Albanian mountain, thus referring to the specific origin of the product. Origin bounded brands (OBBs) are defined as brands that use their origin as their unique selling proposition. Such branding represents an important information stimulus that suggests the unique resources at the product’s core. It should also be noted that the country of origin effects can be different regarding countries and product types [38,77,78], and these are preceded by price choice [79]. Other researchers have shown that consumers associate product origin with other credence attributes, such as food safety [80,81]. In this case, further research and additional methodologies are needed to explore consumers’ expectations, knowledge and the components of preferences concerning brands.

In conclusion, Brand 1 shows brand dominance and recognition compared to the three other brands, indicating brand leadership as a consequence and thus higher BE. However, this is not the only indicator that shows higher brand equity. Response shift is another indicator that helps when judging the value of brands from the consumer perspective. The response shift indicator (F − B) is evident in the cases of Brand 1 and Brand 2, while consumers did not show increasing preference for the other two brands in the full information condition. In full information evaluation, consumers awarded the highest liking scores for the most preferred brands (Brand 1).

The assimilation to expectations indicator was also considered to obtain more information about the BE of the analyzed brands, which expresses brand loyalty by showing if consumers align their expectations in the full information scenarios with the brand scoring situation (F − E = 0). Complete assimilation is shown for Brand 1, as consumers aligned their expectations in the full information situation, showing consumer satisfaction. In the case of Brand 2 and Brand 4, consumers revised their expectations, showing partial positive assimilation for Brand 2 and a negative contrast for Brand 4. This result suggests that a strong brand alone cannot override consumers’ other functional expectations toward the product.

Brand 3 was rated as medium in all three scenarios. The *t*-test demonstrates that the liking score does not change when brand information is introduced or completed.

Results regarding the relative weight of the brand confirm the previous findings—calculation of the assimilation coefficients (see Table 3) shows that Brand 1 has the highest coefficient (β = 0.77; R^2^ = 0.41) compared to Brand 2 (β = 0.6; R^2^ = 0.52) and Brand 4 (β = 0.6; R^2^ = 0.38). The coefficient is higher than 0.5, showing the importance of the brand name weight in product evaluation. Brand 1 is awarded the highest weight, showing high BE. However, the coefficient of determination is higher for Brand 2, so the asymmetrical effect was further analyzed. The prediction is that consumers demonstrate an asymmetrical effect only for the most preferred brand. Researchers usually claim [38,82] that consumers tend to experience negative disconfirmation after higher expectations because higher expectations are more likely to be disconfirmed. According to the results in the case of Brand 2, preliminary expectations based on the brand name were not as high as for the most preferred brand.

Studies show that when dealing with very familiar products such as water, the weight of the brand in the overall product evaluation decreases [52]. However, these findings suggest that even in familiar products, such as mineral water, the brand name plays an essential role in the decision process because it reassures the consumer of their choice and confirmation bias occurs. Confirmation bias tends to interpret new information to become compatible with existing beliefs [83]. Indeed, the results show that consumer evaluations can be associated with an asymmetric disconfirmation effect for the most preferred brand, Brand 1, and in a local brand, where partial positive assimilation is detected (Brand 2). The perceived value of the product is explained by positive disconfirmation behavior. The determination and assimilation coefficients suggest that consumers who negatively disconfirmed awarded a lower weight to the brand, while those disconfirming positively awarded a higher weight to the brand in the product evaluation.

For a one-unit change in the scoring of Brand 1, the perceived value shifts by 0.5 units for the negative disconfirmation score, while a one-unit change in the positive disconfirmation score produces a 0.8 shift in the perceived value of the product. The findings from the EDT indicators for Brand 1 suggest that its communication strategy should reassure customers of their choice when selecting such products. It can be stated that analyzing perceived disconfirmation values is a better indicator in assessing the credence attribute in the overall product evaluation than the total response shift.

Concerning Brand 2, no asymmetrical effect could be observed, although, in the case of a separate response shift, greater variance in the product evaluation was explained by whether the disconfirmation was positive or negative. The assimilation coefficient is higher (from β = 0.6 to β = 0.8) in both cases (see Table 3). The result implies that the consumer highly values the brand and plays an important role in product evaluation. The separate response shift also shows that marketers should focus on improving the taste of their products.

Following the primary outcomes, the paired *t*-test indicates that Brand 3 is the least preferred among the four presented brands (Table 3). In terms of the pair comparisons, Brand 3 is more preferred than Brand 2 but less preferred than Brand 4 in blind and brand information scenarios.

The test results reflect the fact that consumers acknowledged the imported brand. They awarded a higher score to Brand 4 than to the product in the blind test. The labelled origin of a product has been shown to directly and positively impact perceived product safety and indirectly impact purchase intention [84]. The higher score for this brand might be related to consumers’ perception that it is a safer product compared to the others. This opinion may be attributed to the numerous food safety warnings circulating on social media and the lack of trust in Albanian food safety institutions [81]. An increase in awareness of health and safety issues results in a stronger preference for imported brands since, from the consumer perspective, these are produced according to EU standards. In addition, Albanian consumers demonstrate a higher level of trust in European institutions. Other studies have shown that trust in EU food certification schemes may explain the success of foreign products in particular markets [85]. However, when complete information is provided, consumers revise their score for this product by lowering it. Thus, contrast behavior was observed. This result might be associated with customers’ lack of experience with this brand since it is a more expensive, high-end brand and can only be accessed by high-income households. The brand role is important in the overall evaluation of the product, even though the coefficient of determination is the lowest compared to the other brands. Regardless of the revision of expectations, an asymmetrical effect was observed, showing a significant statistical difference between positive and negative disconfirmation. This result might again be linked with consumers’ low confidence levels regarding expectations. Generally, when consumers rely on their expectations and the product does not meet these, the result is a negative disconfirmation, although this is not the case for this brand. The response shift of positive disconfirmation shows a higher determination coefficient and more significant assimilation coefficient than no separate response shift, thus indicating that those who positively disconfirm place greater weight on the brand in the product evaluation.

According to the empirical research, the assimilation coefficient was higher (β = 0.77) for the most preferred brand (Brand 1) than for the others, showing the predominance of the brand from the consumer perspective. In addition, the assimilation coefficient for positive disconfirmation for this brand was higher than that for the other brands; thus, satisfaction is much better explained by positive disconfirmation. This can also be interpreted as confirmation bias for strong brands and may be influenced by the applied experimental setting. However, as a practical implication of the study, it can be concluded that it is important to reinforce consumers in their right choice by developing loyalty programs to maintain satisfaction even for strong brands.

The results confirm the asymmetric disconfirmation effect only for the most preferred brand. Nevertheless, the results also indicate significant statistical differences between positive and negative disconfirmation that may be explained by the low level of consumer confidence regarding expectations.

Consumers did not show any brand preferences or contrast effect for the less preferred brand (Brand 3); this result supports the application of such a methodology in brand evaluation. The observed partial assimilation (Brand 2), contrast (Brand 4), and complete assimilation (Brand 1) suggest the presence of different levels of brand loyalty and equity. Despite the contrast behavior in the imported brand (Brand 4), an asymmetrical effect was detected. Since the response shift of positive disconfirmation shows a higher determination coefficient and a more significant assimilation coefficient, a proportion of consumers are likely attracted to foreign brands. Thus, the brand reputation is paramount to their perception of the product. In their case, brand-building communication through targeted channels can be highly effective from a business perspective.

The level of confidence in expectations moderates the role of expectations in the consumer satisfaction process, thus using the EDT model, Zhand and his co-authors [47] state that disconfirmation harms consumer satisfaction (CS) when expectations are negative, causing negative perceptions. However, Cho et al. [86] show that negative and positive disconfirmation also influences CS. Istianingsih and Defit [87] show that confidence can influence the CS model. With low confidence in expectations, CS is explained mainly by perceived performance, while high confidence implies that CS can be modelled by perceived performance and disconfirmation. Yi and La [88] also posit that higher confidence levels are accompanied by asymmetric disconfirmation.

## 6. Conclusions

The main novelty of this paper is approaching the mineral water purchasing decision process from a sensory perspective, highlighting trust as an important issue in a low involvement product category. Compared to other studies that evaluate trust using attitudes to brand/institutions, by simply asking how much you believe in a particular brand/institution, the present study can differentiate the level of trust between the imported brand, and the origin bounded brand through the combination of EDT and GT. Even though the imported brand showed higher expectations due to higher trust in EU food institutions, by contrasting them, the consumer shows an affinity for the OBB due to the reputation of these brands in Albania. Studies show that OBB creates other intangible services such as cultural ecosystem and environmental ecosystem services [24,89]. Providing these services generates reputation, and the latter creates and reinforces trust. Thus, moving from the Marketing 1.0 approach focused only on product development to Marketing 3.0, focusing on consumer values, will help companies mitigate trust issues even in developing countries. Trust is an essential ingredient to transit toward Marketing 4.0, representing the development of community marketing in the digital area.

The present research suggests that brands can play an essential role in shaping consumer value judgements in a no-trust institutional setting by supporting the customer’s choices and safeguarding the reputation of products in the marketplace.

Combining brand equity indicators with EDT theory provides insight into the hardly measurable influential variables of brand loyalty and brand equity evaluation. The results suggest that the satisfaction level (i.e., product evaluation) can be explained in more depth by the divided response shift indicator in the case of a familiar, low involvement product such as mineral water. It can be stated that the positive and negative disconfirmations of the response shift, which measures the weight of a brand in the overall evaluation of a product, can provide accurate information for brand managers.

### 6.1. Limitations

However, some limitations of the use of EDT in brand asset evaluations need to be considered. Inaccurate expectations can be produced due to a lack of previous experience with products and scores in assessing satisfaction levels. Yüksel and Yüksel [90] state that the dynamic nature of expectations, their meaning to consumers, the use of different scores in assessing satisfaction and product type may result in uncertain and consequently less stable expectations. In this vein, mineral water was intentionally selected as a product that met the purpose of the study, being familiar to consumers (thus, reliable expectations about it were predicted).

From a methodological perspective, EDT indicators should also incorporate external factors such as culture and trust in institutions to produce accurate and reliable results. Expectations are created in the broader context of brands. Thus, adding such related variables to the measurement construct in future studies may be interesting. Analyzing qualitative data with GT could contribute to having a more complex picture of the role of brand equity. Semi-structured interviews could deepen the data complexity. However, the large sample size could not enable such a method.

The model adopted in the research measures brand perception along a single scale using general consumer evaluation, providing useful information for brand strategy development by analyzing the perceived disconfirmation values. The methodology, however, makes it difficult to gain a deeper understanding of consumer preferences and is, therefore, most effective in practice as a complement to other methods.

A further limitation is that Honig et al. [91] showed that the taste of mineral water could be influenced by its ingredients, especially by high HCO_3_^−^ concentration. This study did not include the ingredients of the experimented water. However, this influence was eliminated by tasting the same mineral water in blind and branded conditions.

### 6.2. Practical Implications

As a result of food safety-related issues associated with developing countries, there may be inaccuracies in the brand evaluation management process. The lack of trust in institutional bodies, especially those related to food safety [81], may lead to weak confidence in expectations, potentially resulting in the inefficient use of consumer expectation theory and rendering EDT applications useless. However, the present research suggests that brands can play an essential role in consumer perception in a no-trust institutional setting by supporting the latter’s choices. Brands can mediate product quality and thus support long-term profitability.

To build brand equity in the mineral water category, the origin and food safety issues, such as guarantees and eco-labels, are essential in building trust. However, these, together with emotions, should be emphasized because love marks can engage consumers and build loyalty.

For products that are difficult to distinguish by tasting, such as mineral water, branding is paramount. This result is also confirmed by Carlucci et al. [92], who emphasized that building equity has a notable impact also on the value recognition of the product by customers, and thus the price. This study shows that brands can mediate product quality, thus supporting long-term customer satisfaction and loyalty. Branding may also contribute to product safety, especially in developing countries.

The conducted factors of BE support managerial implications. According to the results, brand recognition is based on brand awareness, supporting the results of Aaker [54] and Farhana [51]. However, this study pays attention also to sensory information and demonstrates that sensory information such as product taste influences consumer perceptions, thus having a notable effect on perceived value. These factors determine customer satisfaction and loyalty; marketers must build their brands based on product quality.

This paper also benefits from assessing how perceived quality differs even regarding familiar brands. The results also indicate that OBBs enhance customer preference toward labels. In the absence of this, international-sounding names are recommended to positively reinforce the quality perceptions, particularly in less developed countries.

Further research should explore other familiar food products to increase the generalizability of the results, as applied to frequently consumed food and beverages. In-depth interviews or focus groups should be conducted to explore more explanatory variables. The impact of non-direct marketing communication tools on perceived quality is an interesting research topic. Combining the brand equity construct with the toolkit on trust measurement in the food sector, including organization trust, food chain trust and product trust, might provide an additional research path with other food products.

## Figures and Tables

**Table 1:** Characteristics of the sample.

Variables	Description	Distribution in %	Mode	Mean	Standard Deviation
**Age category**	1: 18–24 2: 25–34 3: 35–44 4: 45–54 5: 55+	35.5 27.3 13 18.2 6	1 (18–24)	2.3	1.2
**Education**	1: primary school 2: high school 3: undergraduate/graduate	4.3 33.5 62.2	3 (undergraduate/ graduate)	2.5	0.57
**Income EUR/month**	1: EUR 71–214 2: EUR 215–428 3: EUR 429–642 4: EUR 643–857	20.3 50.4 18.7 10.4	2 (215–428)	2.1	0.8

Source: Authors’ construction.

**Table 2:** Brand equity analysis through disconfirmation of expectations indicators.

Brands	Brand (1) Lajthiza (B1)		Brand (2) Tepelena (B2)	Brand (3) Qafshtama (B3)	Brand (4) Acqua Panna (B4)
	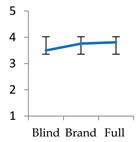		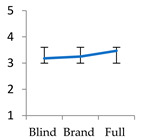	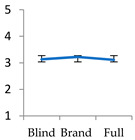	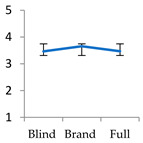
**E − B**	Mean	0.26 *	0.07	−0.09	0.19 *
**F − B**	Mean	0.34 **	0.29 **	ns	ns
**F − E**	Mean	ns	0.21 **	ns	−0.19 *
**Assimilation.** **contrast**	Complete assimilation E − B > 0 F − B > 0 F − E = 0		Partial positive assimilation E − B = 0 F − B > 0 F − E > 0	Contrast not significant	Negative contrast E − B > 0 F − B = 0 F − E < 0
**F − B =** **α + β(E − B) + ε**	R^2^ = 0.41 β = 0.77 *		R^2^ = 0.52 β = 0.6 *	na	R^2^ = 0.38 β = 0.61 *

Note: * Significant at *p* ≤ 0.01 level; ** *p* ≤ 0.001. Note: Expectation score (E); Blind test score (B); Full information test score (F); ns: not significant; na: not applicable; α: Level of assimilation (proportion of the response shift over the degree of incongruence); β, ε: function coefficients. Source: Authors’ construction

**Table 3:** Pair comparisons between brands in the three information situations using paired *t*-test.

Brand Comparisons	Mean Blind	Mean Brand	Mean Full	Brand Comparisons	Mean Blind	Mean Brand	Mean Full
**B3 − B2**	−0.0478 ^ns^	−0.0306 ^ns^	−0.3565 *	**B2 − B4**	−0.2870 *	−0.4061 *	No difference
**B3 − B4**	−0.3348 *	−0.4367 *	−0.3565 *	**B2 − B1**	−0.3217 *	−0.5109 *	−0.3636 *
**B3 − B1**	−0.3696 *	−0.5415 *	−0.6909 *	**B4 − B1**	−0.0348 ^ns^	−0.1048 ^ns^	−0.3545 *

Note: * Significant at *p* ≤ 0.01 level; ns: not significant. Note: B1, B2, B3 and B4 refer to the conducted brands. Source: Author’s elaboration.

## Data Availability

The data presented in the study are available on request form the corresponding author.
